# The adaptor protein PICK1 targets the sorting receptor SorLA

**DOI:** 10.1186/s13041-022-00903-0

**Published:** 2022-02-19

**Authors:** Lars Binkle, Marcel Klein, Uwe Borgmeyer, Dietmar Kuhl, Guido Hermey

**Affiliations:** Institute for Molecular and Cellular Cognition, Center for Molecular Neurobiology Hamburg, University Medical Center Hamburg-Eppendorf, Falkenried 94, 20251 Hamburg, Germany

**Keywords:** SorLA, Sorting receptor, Vps10p-domain, PICK1, PDZ-domain, Protein interaction, Neuronal endosomal sorting and trafficking

## Abstract

SorLA is a member of the Vps10p-domain (Vps10p-D) receptor family of type-I transmembrane proteins conveying neuronal endosomal sorting. The extracellular/luminal moiety of SorLA has a unique mosaic domain composition and interacts with a large number of different and partially unrelated ligands, including the amyloid precursor protein as well as amyloid-β. Several studies support a strong association of SorLA with sporadic and familial forms of Alzheimer’s disease (AD). Although SorLA seems to be an important factor in AD, the large number of different ligands suggests a role as a neuronal multifunctional receptor with additional intracellular sorting capacities. Therefore, understanding the determinants of SorLA’s subcellular targeting might be pertinent for understanding neuronal endosomal sorting mechanisms in general. A number of cytosolic adaptor proteins have already been demonstrated to determine intracellular trafficking of SorLA. Most of these adaptors and several ligands of the extracellular/luminal moiety are shared with the Vps10p-D receptor Sortilin. Although SorLA and Sortilin show both a predominant intracellular and endosomal localization, they are targeted to different endosomal compartments. Thus, independent adaptor proteins may convey their differential endosomal targeting. Here, we hypothesized that Sortilin and SorLA interact with the cytosolic adaptors PSD95 and PICK1 which have been shown to bind the Vps10p-D receptor SorCS3. We observed only an interaction for SorLA and PICK1 in mammalian-two-hybrid, pull-down and cellular recruitment experiments. We demonstrate by mutational analysis that the C-terminal minimal PDZ domain binding motif VIA of SorLA mediates the interaction. Moreover, we show co-localization of SorLA and PICK1 at vesicular structures in primary neurons. Although the physiological role of the interaction between PICK1 and SorLA remains unsolved, our study suggests that PICK1 partakes in regulating SorLA’s intracellular itinerary.

## Introduction

SorLA (also known as LR11 or SORL1) a transmembrane protein with a short cytosolic and a large extracellular/luminal part is prominently, but not exclusively, expressed in the brain [1–3]. It constitutes together with Sortilin, SorCS1, SorCS2 and SorCS3 the vacuolar protein sorting 10 domain (Vps10p-D) receptor family [4, 5]. SorLA’s extracellular/luminal moiety has a unique domain composition. It consists of an N-terminal Vps10p-D, followed by EGF- and complement-type repeats, which are also characteristics of the low-density-lipoprotein receptor (LDLR) family, and fibronectin type-III domains. A large number of different ligands have been described to interact with SorLA. Some neuropeptides and growth factors, such as neurotensin, amyloid-β (Aβ), and ciliary and glial cell line-derived neurotrophic factor bind to the Vps10p-D [6–10]. Other ligands are shared with LDLRs and probably all interact with the cluster of complement-type repeats, including apolipoproteins, lipoprotein lipase, plasminogen activators and the amyloid precursor protein (APP) [1, 8, 11–14]. In addition, SorLA binds and regulates endosomal trafficking of ERBB2 (also termed HER2) [15].

Genetic analysis revealed an association of *SORL1* with both sporadic and familial forms of Alzheimer’s disease (AD) [16]. AD brains show lower expression of SorLA [17], which was also observed in mouse models following amyloidosis [18, 19]. In mice, deletion of SorLA leads to elevated Aβ-levels in the brain and increased SorLA levels go along with a reduced Aβ load [20]. In neurons, SorLA alters the intracellular trafficking of APP and this reduces amyloidogenic processing [14, 20, 21]. Depending on pH, SorLA also binds Aβ in its monomeric form, which probably regulates lysosomal targeting of Aβ [6, 22]. These data substantiate SorLA’s sorting function for APP and Aβ. Although SorLA seems an important factor in AD, the large number of different ligands suggests a role as a multifunctional receptor with additional, APP-independent, cellular functions.

SorLA locates to the TGN and endosomes and only a minority is found on the cell surface [8, 23, 24]. In polarized MDCK cells, SorLA is targeted to the basolateral membrane and to sorting endosomes, in cultured neurons, to the somato-dendritic area [25]. In SorLA deficient hiPSC-derived neurons, altered endosomal trafficking of APP was confirmed, but an overall endosome enlargement was also observed [26, 27]. SorLA ablation seemed to disrupt endosomal cargo processing and cause intracellular traffic jams, supporting a broader role in regulating endosomal transport and sorting [26, 27]. Therefore, understanding the determinants of SorLA’s subcellular itinerary might be pertinent for understanding neuronal endosomal sorting in general.

Cellular uptake of ligands conveyed by SorLA is rather slow when compared to LDLRs such as LRP1 [11]. The cytoplasmic domain contains canonical binding motifs, and the interaction of several cytosolic adaptors has been reported. SorLA interacts with adaptor protein-2 (AP-2), AP-1, GGAs, and retromer [28–31]. It is of note, that these adaptors and many ligands of the extracellular/luminal moiety are shared with the Vps10p-D receptor Sortilin. Although SorLA and Sortilin show both a predominant intracellular and endosomal localization, they are targeted to different endosomal compartments [24, 28]. This indicates differential subcellular sorting of both receptors by exclusive adaptor proteins interacting only with one of the two receptors. So far, the cytosolic adaptor HSPA12A has been shown to target specifically the cytoplasmic domain of SorLA but not of Sortilin [32].

To date, only a few adaptor proteins have been described to bind the cytosolic domains of the SorCS subset of Vps10p-D receptors. SorCS1 interacts with AP-2 that likely mediates the internalization of all Vps10p-D receptors [33]. The SorCS3 cytosolic domain binds the PDZ (postsynaptic density-95/disc-large/zona-occludens-1) domain proteins PSD95 and PICK1 [34]. Here, we hypothesized that Sortilin and SorLA share these interactions. We assessed the binding of PSD95 and PICK1 to Sortilin and SorLA and observed an interaction only for SorLA and PICK1.

PICK1 has a unique domain composition with a single N-terminal PDZ domain followed by a BAR (Bin-Amphiphysin-Rvs) domain [35, 36]. PICK1 has been first described as an interactor and substrate of protein kinase C [37]. In addition, the PDZ domain mediates binding to the C-termini of many transmembrane proteins, ion channels and kinases, including the AMPA receptor subunit GluA2, mGluR7, monoamine transporters, ADP ribosylation factors, and ERBB2 [36, 38–44]. The BAR domain is a protein module thought to induce and stabilize lipid membrane curvature [45]. In agreement, an amphipathic helix N-terminal to the PICK1 BAR domain conveys membrane curvature sensing [46]. Initial studies demonstrated a prominent perinuclear localization of PICK1 [37]. Subsequent studies demonstrated localization of PICK1 to Golgi compartments and secretory vesicles [47, 48]. It plays a critical role in the budding of immature secretory vesicles from the TGN [48], and in secretory vesicle biogenesis [47, 49]. In neurons, PICK1 localizes to pre- and postsynaptic membranes [38, 50, 51]. It controls activity-regulated synaptic endosomal cargo retrieval at presynaptic nerve terminals [50]. At postsynaptic membranes, PICK1 binds GluA2 and is participating in NMDA receptor-induced calcium-dependent internalization of AMPA receptors, a critical process for multiple forms of synaptic plasticity [35, 52–57].

The PICK1 PDZ domain tethers transmembrane proteins as cargo and the BAR domain binds curved-membranes which can bud and form transport vesicles. It becomes fully functional through homo- or hetero-dimerization with another BAR domain protein. PICK1 constitutes a heteromeric BAR-domain complex with ICA69 [47, 48]. These complexes are mainly localized to the TGN, on budding immature dense core vesicles containing either proinsulin (in pancreactic beta cells) or growth hormone (in pituitary cells). In contrast, PICK1-positive but ICA69-negative complexes that are likely PICK1 homomeric complexes are found at mature secretory granules [47, 48]. Moreover, a change from PICK1-ICA69 heterodimers to PICK1 homodimers has been suggested to control the neuronal trafficking of AMPA receptors to synapses [58].

Although the physiological significance of the interaction of PICK1 and several of its ligands remains poorly understood, PICK1 regulates the trafficking of transmembrane proteins through binding their C-termini with its PDZ domain.

## Results

**Fig. 1:**
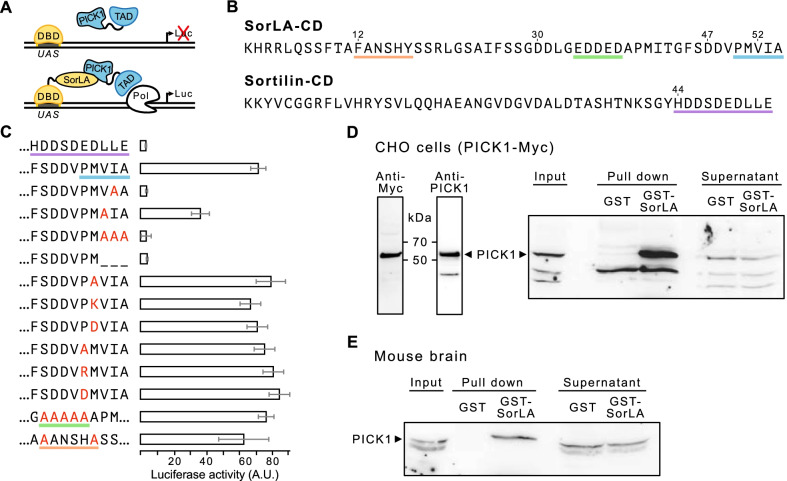
SorLA interacts with PICK1. **A** Principle of the mammalian-2-hybrid system. DBD, DNA-binding domain; Luc, firefly luciferase; Pol, polymerase; TAD, transcription activation domain; UAS, upstream activating sequence. **B** Primary sequence of the SorLA cytoplasmic domain (CD) (top) and of the Sortilin cytoplasmic domain. Protein interaction motifs mutated in (**C**) are underlined. **C** Mammalian-2-Hybrid analysis of PICK1 with the cytoplasmic domain of Sortilin or SorLA. Co-transfections of N2a cells with fusion constructs of the GAL4 DNA binding domain with wild-type Sortilin or SorLA or the mutant cytoplasmic domain of SorLA and a fusion construct of the VP16 transcriptional activation domain with full length PICK1 were analyzed. Terminal or mutated amino acid sequences of the cytoplasmic domains are indicated on the left. Motifs marked in (**B**) are indicated with the same color code. Luciferase activity was normalized to the fluorescence generated by a co-transfected eGFP vector. Relative luciferase activity (mean ± SD) based on a typical experiment performed in septuplicate. **D** Detection of PICK1-Myc in stably transfected CHO-cells by immunoblotting using an anti-Myc or anti-PICK1 antibody (left). Pull-down of PICK1-Myc from stably transfected CHO-cells (right). Precipitation was performed with GST or GST fused to the cytoplasmic domain of SorLA and analyzed by immunoblotting using an anti-Pick1 antibody. **E** Pull down of PICK1 from murine brain lysates. GST or GST fused to the cytoplasmic domain of SorLA was used to pull down from brain lysates and analyzed by immunoblotting using an anti-PICK1 antibody

We investigated the potential interaction of the cytoplasmic domains of SorLA and Sortilin with PICK1 by mammalian-2-hybrid analysis (Fig. 1A, B). The mammalian 2-hybrid system is based on the yeast DNA-binding domain (DBD) of GAL4 and the transcription activation domain (TAD) VP16 of the herpes simplex virus. Both domains are separated and reconstitution occurs only by interaction of fused proteins (Fig. 1A). When reconstituted, DBD binds to an upstream activating sequence (UAS) and TAD drives firefly luciferase (Luc) expression from a reporter plasmid. To analyze the interaction of SorLA and Sortilin with PICK1, we fused the cytoplasmic domain of SorLA as well as of Sortilin with the DBD and fused PICK1 with the TAD (Fig. 1A). The luciferase activity of transfected N2a cells showed binding of PICK1 to the cytoplasmic domain of SorLA (SorLA-CD), but not to the cytoplasmic domain of Sortilin (Sortilin-CD) (Fig. 1B, C). To demonstrate the specificity of the interaction, we generated SorLA-CD mutants and analyzed their binding capabilities to PICK1 (Fig. 1C). PICK1 binding was abolished by mutating I^53^ or V^52^ and I^53^ to alanine (Fig. 1C). Mutation of only V^52^ showed an attenuated interaction. There was no detectable PICK1 binding when the C-terminal amino acids were removed. Mutations of M^51^ or P^50^ to alanine or to a basic or acidic amino acid did not affect the binding. Moreover, mutations of the pentameric acidic cluster E^34^-D^38^ to AAAAA (Fig. 1B, C indicated in green), which is essential for interaction with AP-1 and AP-2 [28] as well as alanine mutations of the retromer interaction motif FANSHY to AANSHA [29] (Fig. 1B, C indicated in orange) did not alter the binding. These analyses demonstrate an interaction of PICK1 with the C-terminus of SorLA-CD of which the three C-terminal amino acids appear critical for binding. To corroborate the observed interaction, we performed GST pull-down experiments. We stably transfected CHO cells with Myc-tagged PICK1 and confirmed the expression through immunoblot analysis using anti-Myc and anti-PICK1 antibodies demonstrating the specificity of the applied anti-PICK1 antibody (Fig. 1D). A fusion protein of GST and SorLA-CD but not GST alone pulled down PICK1-myc from lysates of the stable transfectants (Fig. 1D). Subsequently, we used GST and GST-SorLA to pull-down proteins from mouse brain extracts and PICK1 was detected only in the GST-SorLA fraction (Fig. 1E).

**Fig. 2:**
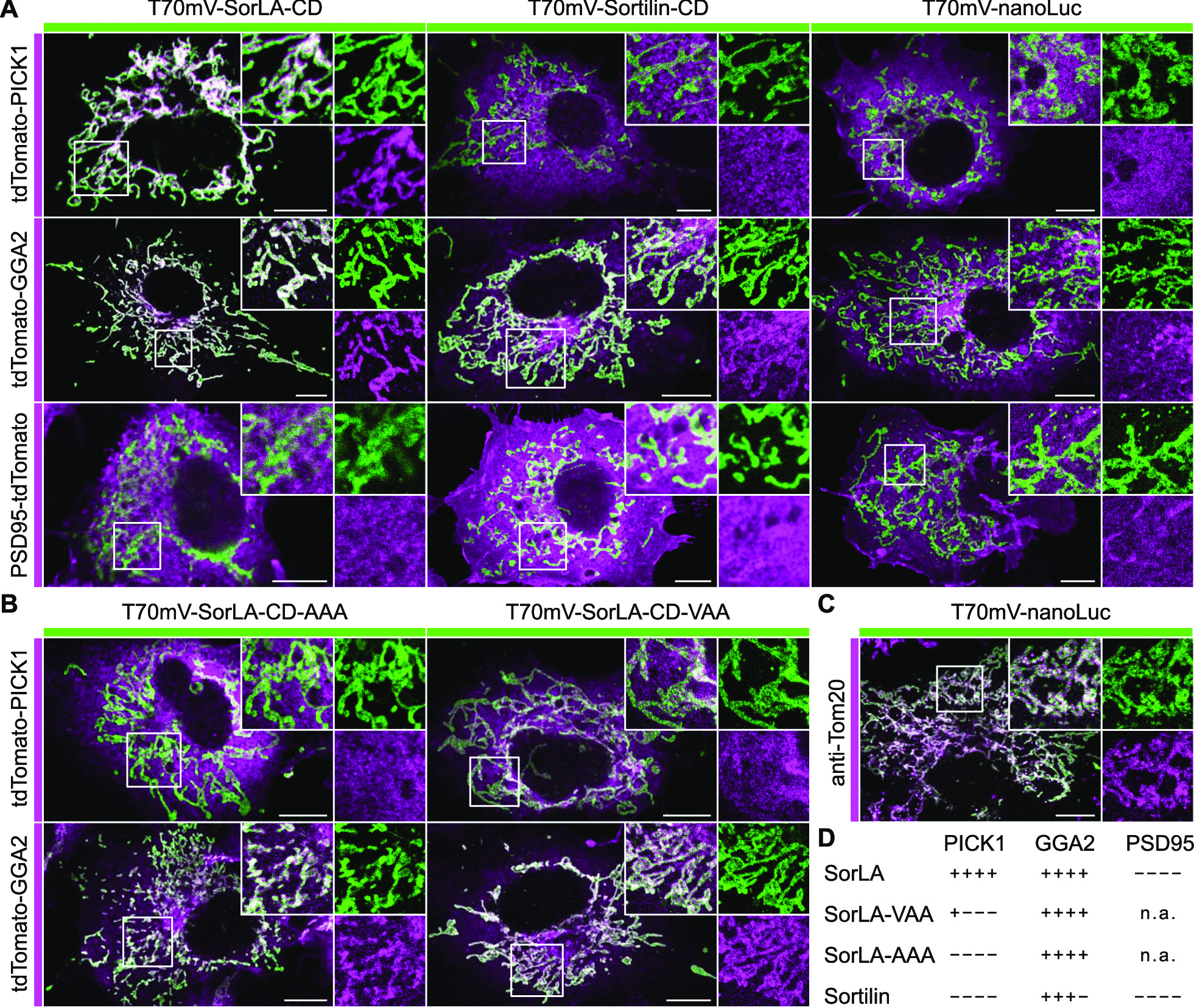
Ectopically localized SorLA cytoplasmic domain recruits PICK1. **A** COS7 cells were transfected with T70mV-SorLA-CD, T70mV-Sortilin-CD or T70mV-nanoLuc (green) and co-transfected with tdTomato-PICK1, tdTomato-GGA2 or PSD95-tdTomato (magenta) as indicated. Magnifications of selected areas (boxes) are shown as insets. mVenus and tdTomato signals were enhanced by immunostainings with respective antibodies. **B** COS7 cells were transfected with the mutated SorLA constructs T70mV-SorLA-CD-AAA or T70mV-SorLA-CD-VAA (green) and co-transfected with tdTomato-PICK1 or tdTomato-GGA2 (magenta) as indicated. **C** Ectopic localization of T70mV-nanoLuc at mitochondria. COS7 cells were transfected with T70mV-nanoLuc (green) and stained with an antibody against the mitochondrial protein Tom20 (magenta). **D** Summary of the recruitment of the tdTomato fusion constructs of PICK1, GGA2 and PSD95 by the analyzed cytoplasmic domains to mitochondria. Scale bars: 10 µm

**Fig. 3:**
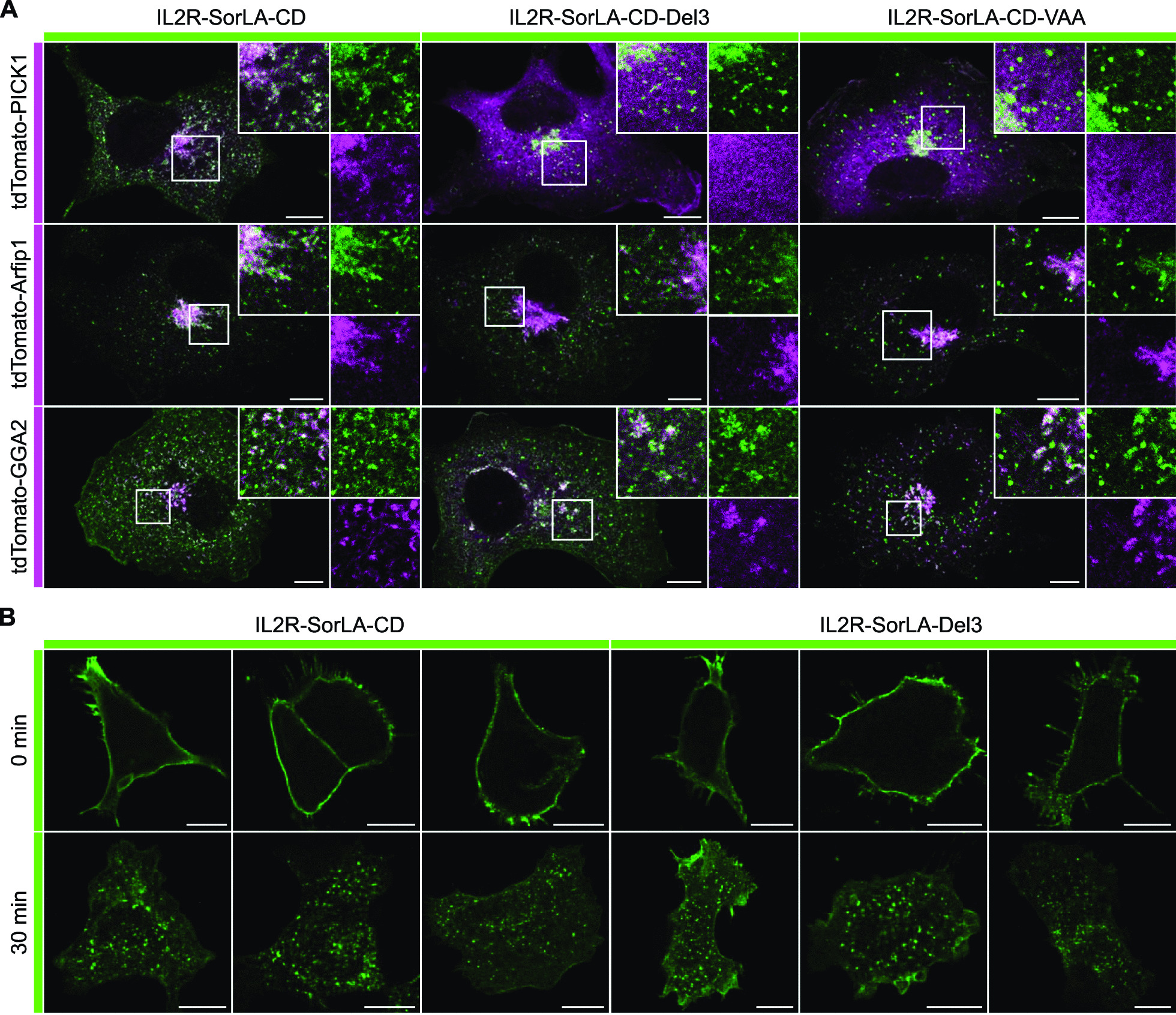
The extreme C-terminus of SorLA recruits PICK1 but is dispensable for internalization. **A** COS7 cells were transfected with IL2R-SorLA-CD, IL2R-SorLA-CD-Del3, IL2R-SorLA-CD-VAA (green) and co-transfected with tdTomato-PICK1, tdTomato-Arfip1 or tdTomato-GGA2 (magenta) as indicated. Magnifications of selected areas (boxes) are shown as insets. IL2R-SorLA and tdTomato constructs were stained with anti-IL2Rα and anti-tdTomato respectively. **B** SY5Y cells were transfected with IL2R-SorLA-CD and IL2R-SorLA-CD-Del3. Cells were incubated with anti-IL2Rα at 4 °C, fixed after 0 or 30 min at 37 °C to allow internalization and immunostained. Three cells per time point and transfected construct are shown. Scale bars: 10 µm

To study the interaction in a physiological cellular environment, we ectopically expressed the SorLA-CD or Sortilin-CD in COS7 cells and analyzed subsequent recruitment of PICK1, GGA2 or PSD95 to the ectopic site. GGA2 has been shown before to interact with Sortilin and SorLA [30, 59]. Ectopic expression of the SorLA and Sortilin cytoplasmic domains on mitochondria was achieved by their fusion to the first 88 amino acids of the yeast mitochondrial protein Tom70 (T70). The fluorophore mVenus (mV) was inserted between T70 and the cytoplasmic domains (CD) for visualization and these constructs were named T70mV-SorLA-CD and T70mV-Sortilin-CD respectively. We used the fusion of nanoLuc luciferase (nanoLuc) to T70mV as a negative control. Co-expression of T70mV-SorLA-CD and tdTomato-PICK1 or tdTomato-GGA2, but not of PSD95-tdTomato, resulted in the recruitment of tdTomato tagged proteins to mitochondria (Fig. 2A). T70mV-Sortilin-CD recruited tdTomato-GGA2, but not tdTomato-PICK1 or PSD95-tdTomato. Co-expression of T70mV-nanoLuc with tdTomato-PICK1, tdTomato-GGA2 or PSD95-tdTomato led to no recruitment and a diffuse tdTomato staining was observed (Fig. 2A). However, the ectopic expression of T70mV-nanoLuc on mitochondria was confirmed by immunostaining for the mitochondrial marker protein Tom20 (Fig. 2C). These results confirm the interaction of SorLA-CD with GGA2 and PICK1 whereas Sortilin-CD interacts with GGA2 but not with PICK1. Mutating the last three C-terminal amino acids of SorLA from VIA to AAA in T70mV-SorLA-CD abolished the recruitment of tdTomato-PICK1 (Fig. 2B). Mutating the second last amino acid of the SorLA-CD (I^53^) to alanine (T70mV-SorLA-CD-VAA) strongly reduced, but did not impede completely the recruitment of tdTomato-PICK1 (Fig. 2B). None of these mutations affected the recruitment of tdTomato-GGA2 to mitochondria (Fig. 2B). Figure 2D summarizes the results of these analyses. Next, we asked if the SorLA-CD fused to a transmembrane domain of a type-I transmembrane receptor recruits PICK1 in COS7 cells which do not express PICK1. To this end, we expressed a chimeric receptor composed of the extracellular and transmembrane moiety of the interleukin 2 receptor alpha (IL2R) fused to the SorLA-CD (IL2R-SorLA-CD). This construct was expressed together with tdTomato-PICK1 in COS7 cells and we observed co-localization of both proteins to perinuclear and vesicular structures (Fig. 3A). Expression of constructs with a deletion of the three terminal amino acids of SorLA (IL2R-SorLA-CD-Del3) or mutation of the second last amino acid of SorLA (I^53^) to alanine (IL2R-SorLA-CD-VAA) resulted in a similar subcellular localization as compared to IL2R-SorLA-CD (Fig. 3A). However, both did not co-localize with tdTomato-PICK1, which shows a diffuse distribution (Fig. 3A). These results suggest that expression of IL2R-SorLA-CD leads to perinuclear and vesicular localization of the interacting tdTomato-PICK1 in cells with no endogenous PICK1 expression. In contrast, the subcellular localization of GGA2 and of the trans-Golgi marker protein Arfip1 expressed as tdTomato fusion proteins appeared not altered in cells co-expressing IL2R-SorLA-CD mutants as compared to the wild type construct (Fig. 3A). These experiments demonstrate the interaction of SorLA-CD and PICK1 under physiological conditions in cells. They also corroborate the results obtained by mammalian-2-hybrid analysis, showing that the interaction of PICK1 with SorLA-CD depends on the three C-terminal amino acids.

A previous study showed that the truncation of the terminal four amino acids of SorLA (M^51^-A^54^) does not change its internalization rate in CHO cells [28]. We used SY5Y cells that express PICK1 endogenously to determine its role in SorLA internalization. PICK1 interacts with the three C-terminal amino acids of SorLA-CD. If PICK1 plays a prominent role in SorLA internalization, deletion of the three terminal amino acids of SorLA should impede its endocytosis. We expressed IL2R-SorLA-CD and the mutant IL2R-SorLA-CD-Del3 in SY5Y cells, incubated cells at 4 °C with an anti-IL2R antibody, washed the cells and incubated the cells for 0 or 30 min at 37 °C (Fig. 3B). Without incubation at 37 °C, IL2R-SorLA-CD and IL2R-SorLA-CD-Del3 showed a prominent surface localization. After incubation at 37 °C for 30 min, both constructs were detected to a similar degree in intracellular vesicular structures. As we did not detect any differences between the uptake of the two constructs, the results strongly suggest that the interaction of PICK1 with SorLA-CD does not mediate internalization of SorLA.

**Fig. 4:**
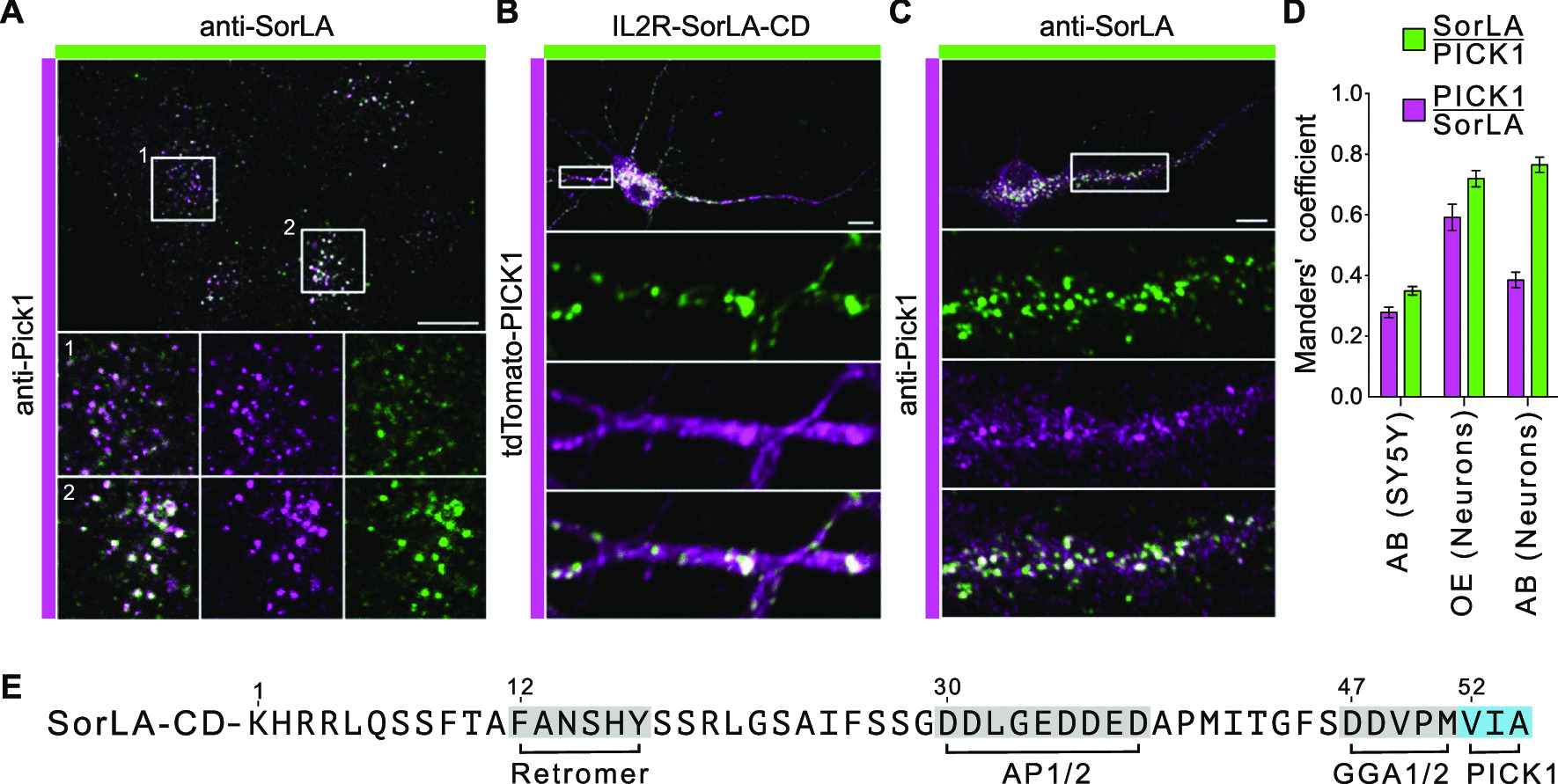
SorLA and PICK1 co-localize. **A** SY5Y cells were stained with anti-PICK1 (green) and anti-SorLA (magenta) antibodies. Magnifications of two selected areas (boxes) are shown. **B** Immunocytochemical localization of IL2R-SorLA and tdTomato-PICK1 in transfected dissociated primary cultured hippocampal neurons using anti-IL2R (green) and anti-tdTomato (magenta) antibodies. Magnification of a selected area (boxes) are shown. **C** Immunolocalization of endogenous SorLA and PICK1 in dissociated primary cultured hippocampal neurons using anti-SorLA (green) and anti-PICK1 (magenta) antibodies. Magnification of a selected area (boxes) are shown. **D** Respective Manders’ coefficient of SorLA and PICK1 using antibodies (AB) against the endogenous proteins or of overexpressed (OE) IL2R-SorLA and tdTomato-PICK1. **E** Primary sequence of the SorLA cytoplasmic domain. Amino acid motifs mediating interaction with retromer, AP-1 and AP-2 and GGA1 and GGA2 are indicated in grey. The here described PICK1 interaction motif is indicated in blue. Scale bars: 10 µm

In order to assess localization of endogenous SorLA and PICK1, we immunostained SY5Y cells using antibodies against PICK1 and SorLA and observed co-localization at larger vesicular structures (Fig. 4A, D). We expressed IL2R-SorLA-CD together with tdTomato-PICK1 in dissociated primary cultured hippocampal neurons, which have no endogenous expression of IL2R. Immunocytochemical analysis demonstrated for both proteins a predominant somatic distribution that extended into neurites. We observed co-localization to a large number of vesicular structures in the soma and in proximal neurites (Fig. 4B, D). Finally, we immunostained dissociated primary cultured hippocampal neurons for endogenous SorLA and PICK1 and observed colocalization at endosomal structures in the soma and proximal parts of neurites (Fig. 4C, D).

## Discussion

Here, we show an interaction of PICK1 with SorLA mediated by the last three C-terminal amino acids, VIA, of SorLA. This corresponds to a type II PDZ domain interaction motif. The canonical classification of PDZ domain interactions with C-termini assorts four major classes; I interacts with (S/T)-X-Φ, II interacts with Φ-X-Φ, III interacts with Ψ-X-Φ, and IV which interacts with (D/E)-X-Φ as preferred C-terminal motif, where X is any residue, Φ is a hydrophobic residue and Ψ is a basic hydrophilic residue [60]. Most PDZ domains interact with only one classified sequence type. In contrast, the PICK1 PDZ domain belongs to a small group of promiscuous PDZ domains with a mixed specificity. PICK1 binds sequences of class I and II interaction motifs and also some atypical motifs [61, 62]. Thus, it is highly likely that the PICK1 PDZ domain mediates the interaction with SorLA-CD. Another PICK1 interactor is ERBB2, which is also forming a complex with SorLA [39]. It is tempting to speculate that a SorLA ERBB2/HER2 complex recruits PICK1, which homodimerizes through its BAR domain and facilitates endosomal transport of the complex.

Our study adds PICK1 to the portfolio of cytosolic SorLA interacting proteins. In general, short linear amino acid motifs mediate the binding of cytosolic domains of transmembrane proteins to adaptor proteins. A number of previous studies addressed the interaction of SorLA-CD with adaptor proteins, identified specific amino acid motifs that mediate the interaction and analyzed the consequences of their mutation or deletion for the subcellular localization of SorLA. The cytoplasmic domain of SorLA interacts through the F^12^ANSHY^17^ motif with retromer [29], the acidic cluster D^30^–D^38^ facilitates binding of AP-1, AP-2 and PACS1, the heat shock protein HSPA12A binds the SorLA-CD via the acidic residues E^34^-D^38^ and D^47^D^48^ in an ADP/ATP-dependent manner and D^47^DXXM is regarded as the minimal GGA interaction motif (Fig. 4E) [28, 30–32]. Accordingly, the here identified PICK1 binding site is located adjacent to the minimal GGA binding motif (Fig. 4E).

Partial ablation or mutation of the retromer interaction motif in SorLA-CD caused endosomal accumulation of SorLA, altered APP sorting and increased APP processing [29, 63]. Additional mutational analysis revealed that the pentameric acidic cluster (E^34^-D^38^) is crucial for SorLA’s internalization, TGN to endosome transport and polarized neuronal targeting [25, 28]. SorLA’s anterograde TGN to endosome transport relies on the acidic cluster in combination with the minimal GGA-binding motif D^48^XXM^51^ [28]. Here, we observed unaltered uptake of a chimeric construct in SY5Y cells after deletion of the terminal three amino acids (V^52^-A^54^). Truncation of the terminal four amino acids (M^51^-A^54^) does also not change the internalization rate in CHO cells [28] nor the polarized sorting of SorLA in neurons [25]. However, the subcellular distribution of such a mutant is changed towards a reduced TGN and increased endosomal localization as compared to the wild-type receptor [28, 31]. Taken together, we demonstrate that SorLA-CD interacts with PICK1 via its three C-terminal residues (VIA). In addition, a function of the last four amino acids in the transport of SorLA between Golgi and endosomes has already be shown. Considering the role of PICK1 in endosomal transport, it is likely that PICK1 is capable of regulating SorLA’s intracellular itinerary.

## Methods

### Antibodies and DNA constructs

The following primary antibodies were used: rabbit anti-dsRed (tdTomato) (632496, Clontech Takara); chicken anti-GFP (mVenus) (ab139701, Abcam); mouse monoclonal anti-interleukin receptor 2 alpha (IL2R-alpha, Tac/CD25) (ab8235, Abcam); mouse monoclonal anti-Myc – 9E10 (MMS-150P, Covance); rabbit polyclonal anti-PICK1 (ab3420, Abcam); mouse monoclonal anti-PICK1 (MABN75, Millipore); rabbit anti-SorLA (SorLA-Vps10p domain) (a gift from C.M. Petersen, Aarhus University, Denmark) [8]; rabbit anti-Tom20 (sc-11415, Santa Cruz Biotechnology Inc.). As secondary antibodies, fluorescent Alexa Fluor-conjugated (Invitrogen) or horseradish peroxidase (HRP)-conjugated (Dianova) antibodies were used.

The construct of the SorLA-cd inserted in pGEX4T-1 (Amersham Pharmacia) has been described before [28]. To generate expression constructs encoding PICK1-Myc, the PICK1 cDNA was cloned into pcDNA4/Myc-His (Invitrogen). To generate VP16 fusion proteins, PICK1 or PSD95 cDNA was cloned in frame via BamHI and NotI into pAct (Promega). To express GAL4 fusion proteins cDNA encoding wild-type or mutant cytoplasmic domains of SorLA or Sortilin were cloned in frame via BamHI and NotI into pBind3-D [64]. Mutations in the amino acid sequence of the SorLA cytoplasmic domain were introduced by PCR using appropriate primers. To recruit the SorLA or the Sortilin cytoplasmic domain to mitochondria, a modified pFUGW vector was used harboring a CMV promoter and a multiple cloning site (Binkle, unpublished). T70mV-SorLA-cd or T70mV-Sortilin-cd were generated by inserting the coding sequence of the first 88 amino acids of yeast Tom70 (T70) followed by the fluorophore mVenus (mV) and the cytoplasmic domains of SorLA, its mutant sequence, or Sortilin respectively. NanoLuc luciferase (nanoLuc) was cloned via PCR using pNL1.1 (Promega) as template and together with T70mV into the modified expression vector. To tag PICK1, PSD95, GGA2 and Arfip1 with the fluorophore tdTomato respective coding sequence was cloned into the expression vector L21C-Nt-tdTomato (Binkle, unpublished). GGA2 was cloned from pcDNA3-Flag-HA-GGA2 (obtained from W. Sellers through Addgene). The chimeric receptor construct (IL2R-SorLA-CD) corresponds to the extracellular and transmembrane domains of the interleukin-2 receptor alpha and SorLA’s cytoplasmic domain or mutated versions of the SorLA cytoplasmic domain. Fragments were PCR amplified and cloned into pcDNA3.1/Zeo (Invitrogen).

### Cell culture

CHO and COS7 cells were cultured in DMEM (Invitrogen) supplemented with 10% (v/v) fetal calf serum (FCS). SH-SY5Y cells were cultured in DMEM (Invitrogen) supplemented with 20% (v/v) fetal calf serum (FCS). Murine neuroblastoma N2a (Neuro-2a) cells were grown in Opti-MEM™ without Phenol Red (Invitrogen) supplemented with 5% (v/v) FCS. All cells were cultured at 37 °C and 5% CO_2_. Lipofectamine™ 2000 (Invitrogen) was used for transfection of cells. Generation of stable transfected cells was described before [65]. Dissociated hippocampal neurons were prepared and cultured as described before [66]. Animal husbandry was authorized under German regulations on animal welfare in accordance with the European Communities Council Directive (2010/63/EU). After 3 days in vitro, neurons were transfected with Lipofectamine® LTX (Invitrogen) and immunostained at 14 days in vitro.

### Mammalian two-hybrid analysis

Interaction of proteins was determined using a modification of the Checkmate mammalian two-hybrid system (Promega). Two vectors were used, pAct and pBind3-D. pAct (Promega) contains the herpes simplex virus VP16 activation domain followed by a multiple cloning site. pBind3-D [64] is a modification of pBind (Promega) in which the DNA-binding domain of the yeast GAL4 gene followed by an altered multiple cloning site and the vector lacks the *Renilla* luciferase module.

N2a cells were seeded on 96-well plates and transfected in parallel with pAct, pBind3-D, peGFP (Clontech), and a pG5luc (Promega) expressing firefly luciferase under control of GAL4 [64]. The next day fluorescence generated by eGFP was determined for normalization and light emission generated by luciferase activity was detected after adding Bright-Glo (Luciferase Assay System, Promega) with a Multilabel Counter (PerkinElmer). Luciferase activity was normalized to eGFP-fluorescence. All transfections and analysis were performed in septuplicate and experiments repeated three times. Average relative luciferase light units and S.D. were determined using the Prism software.

### Pull down analysis

GST or GST-fusion proteins were expressed in BL21(DE3) cells, and purified by using glutathione-sepharose beads according to the manufacturer’s recommendations (Amersham Pharmacia). Stable transfected CHO cells expressing PICK1-Myc were lysed in 150 mM NaCl, 1% Triton X-100, 20 mM Tris–HCl, 10 mM EDTA, pH 8.0 supplemented with protease inhibitors (Complete Mini, Roche), cell debris sedimented for 20 min at 16.000 g and 10% of each supernatant was used as an expression control (input). Supernatants were incubated with 30 µl Pierce™ glutathione magnetic agarose beads (ThermoFisher Scientific) prebound with GST or GST-SorLA-cd for 2 h at 4 °C. Subsequently, protein complexes were subjected to immunoblotting.

For pull down of endogenous proteins, brain homogenates were prepared from 2 mouse forebrains in 320 mM Sucrose, 4 mM Hepes, 2 mM MgCl, 2 mM CaCl, pH 7.5 supplemented with protease inhibitors (Complete Mini, Roche) using a glass homogenizer (12 passes) (modified from [38]). Homogenates were centrifuged at 1000 g for 10 min, and the supernatant (S1) was centrifuged at 48,000 g for 30 min to obtain the pellet (p2) fraction. This fraction was resuspended in 0.1 mM EDTA, 1% Triton, pH 7.4 plus protease inhibitor, sonicated and solubilized for 1 h at 4 °C. After centrifugation at 100,000 g for 1 h the supernatant (S3) was used for GST pull-down assays. GST fusion proteins coupled to Pierce™ glutathione magnetic agarose beads were incubated overnight, washed with PBS, 0.1 mM EDTA, 0.1% Triton, pH 7.4 plus protease inhibitor and analyzed by immunoblotting as described before [65].

### Immunocytochemistry and internalization assay

Cells were cultured on coverslips, fixed, permeabilized and stained as described before [65]. To visualize internalization, cells were surface labeled with primary antibody (anti-IL2Rα) at 4 °C for 1 h followed by fixation or incubation at 37 °C for 30 min, fixation and staining with secondary fluorescent antibody. All immunocytochemical stainings were performed in triplicates. Cells were analyzed by confocal microscopy using a Leica TCS SP8 microscope and a 63x (NA = 1.4) oil immersion objective.

## Data Availability

All data generated or analyzed during this study are included in this article. Materials are available from the corresponding author on reasonable request.

## Abbreviations

Aβ: Amyloid-β
AD: Alzheimer’s disease
APP: Amyloid precursor protein
ERBB2: Epidermal growth factor receptor 2
DBD: DNA-binding domain
hiPSC: Human induced pluripotent stem cell
IL2R: Interleukin 2 receptor alpha
LDLR: Low-density-lipoprotein receptor
Luc: Firefly luciferase
SorLA-CD: Cytoplasmic domain of SorLA
Sortilin-CD: Cytoplasmic domain of Sortilin
TAD: Transcription activation domain
TGN: Trans-Golgi-network
Vps10p-D: Vacuolar protein sorting 10 domain
UAS: Upstream activating sequence
